# Regional Variation of Suicide Mortality in South Korea

**DOI:** 10.3390/ijerph17155433

**Published:** 2020-07-28

**Authors:** Minjae Choi, Yo Han Lee

**Affiliations:** 1 Department of Public Health, Korea University, Seoul 02841, Korea; choiminjae@korea.ac.kr; 2 Graduate School of Public Health, Ajou University, Suwon 16499, Korea

**Keywords:** suicide rate, regional variation, financial independence, social welfare budget

## Abstract

South Korea’s suicide rate is the highest among the members of the Organization for Economic Cooperation and Development. This study seeks to verify regional variation in suicide rates in South Korea and to identify correlating factors. We used age-adjusted suicide rates for 252 administrative districts, and a Community Health Survey, national representative data, and other national representative data such as censuses were used to obtain information on socioeconomic, health related and social integration variables according to each administrative district. Regional variation in suicide rates was analyzed by using Extremal Quotient (EQ), and multiple linear regression analyses were used to investigate associations between variation in suicide rates and regional socioeconomic, public service factors and health related factors. The average suicide rate from 252 regions was 142.7 per 100,000 people. The highest region was Hongchun-gun (217.8) and the lowest was Gwachen-si (75.5). The EQ was 2.89, meaning that there is significant regional variation in suicide rates. Financial independence (β = −0.662, *p* < 0.001), social welfare budget (β = −0.754, *p* < 0.001) and divorce rates (β = 17.743, *p* < 0.001) were significant, along with other adjusted variables. This study suggests considering these factors in order to reduce suicide rates in South Korea.

## 1. Introduction

According to the World Health Organization (WHO), suicide is defined as the act of deliberately killing oneself with knowledge of the consequence of the act [1]. Worldwide, the number of suicides is over 800,000 annually, which may be age-adjusted to 11.4 per 100,000 people [2]. The WHO is aware of this situation, and has made a plan to reduce suicide rates by 10% by 2020 [3]. In an Irish study, the economic cost of suicide was shown to be about 830 million euro, i.e., 1% of the nation’s gross national product [4]. Another study showed that the burden of suicide relative to the total burden of all diseases increased from 1.8% in 1998 to 2.4% in 2020 [5]. Regarding public health, the pain of those bereaved by suicide does not disappear, but is transferred to family, friends and even communities [6]. As many researchers are aware of the seriousness of suicide, numerous studies of risk factors have been carried out.

A wide range of risk factors for suicide have been identified, related to distal factors, such as socioeconomic and cultural factors, and proximal factors, such as psychiatric disorders and physical illnesses [7]. As for socioeconomic status, the lower the education level, income and social class, the higher the risk of suicide [7,8,9]. Regarding physical and psychiatric disorders, many studies have found that not only a single disease, but also multimorbidity conditions are related with suicide [7,10,11]. Empirical studies support that mental disorders such as substance abuse, schizophrenia and psychosis are associated with suicide [12,13]. Demographic factors such as older age and male sex are also related to suicide [14]. However, these studies identified an association between suicide and various risk factors at the individual level only.

Suicide is influenced by social factors that give rise to circumstances changing human behavior or making people more psychologically or physically unhealthy, which induce suicide or suicidal ideation [15]. So, the circumstances affecting people at the regional or community levels, and not at the individual level, need to be clarified. Durkheim developed a theory that attributed variations in suicide rates to different characteristics in different societies [16]. Since suicide has different meanings in different societies and circumstances, its patterns vary according to region [17]. Among various regional characteristics, many sociocultural factors contribute to regional variations in suicide rates [18]. In recent years, studies dealing with risk factors at the social level have attracted attention [18,19,20,21,22]. This trend has developed spatial clustering techniques which identify geographic patterns and associated characteristics across clusters [23].

Several studies have been conducted on suicide at the regional level. Concerning demographic factors, population density, marriage status, moving status, region, single parents and race have been associated with suicide rates [20,24,25,26,27]. Regarding socioeconomic factors, employment status and average income were shown to affect regional suicide rates [20,27]. For health-related factors, alcohol-related psychiatric illness is related with suicide rates in Slovenia [24]. To identify these factors, there are various methodologies such as regression analysis models, the principal component analysis model, geographic weighted regression model and Bayesian hierarchical models [20,25,26,27]. However, these studies lacked adequate degrees of variation of suicide rate and various factors. Meanwhile, few studies have been undertaken on variations in suicide rates among regions, and the few that have been published included socioeconomic factors only [26,27,28]. Even though various factors have been used, studies of suicide at the regional level should also focus on environmental and population aspects, such as social welfare budget [14].

In South Korea, the suicide mortality rate has been the highest among the members of Organization for Economic Cooperation and Development (OECD) since 2003, showing almost double the OECD average. Thus, more comprehensive understanding of sociodemographic, economic, public service and health related factors is needed to construct more effective strategies for the prevention of suicide in South Korea. The aim of this study is to verify regional variations in suicide rates and to identify associated factors at the regional level.

## 2. Materials and Methods 

### 2.1. Study Design and Data Source

This study is an ecological study, in which the study unit is a population [29]. Data for this study were obtained from the 2010 Community Health Survey data, the National Death Registration from 2010–2014 and other administrative data from South Korea from 2010. The Community Health survey has been conducted annually since 2008 by the Korea Center for Disease Control and Prevention (KCDC) [30]. The purpose of this survey is to assess health conditions, health-related behavior and socioeconomic factors of the Korean population and to produce community health statistics [30,31]. In South Korea, Statistic Korea provides regional level unit information such as age-adjusted specific mortality and other sociodemographic and economic factors. These data were obtained from the National Census survey data, National death registration data and other administrative information. So, we assumed that these data were representative of the specific, Korean context which is the focus of this study. Since this information was included in a website analysis, we could access regional level information using the web-quarry system. Region-based information has different population units and we needed to unify cities because data from various data sources provided different population units. We divided the region of South Korea into 252 cities. The study was exempted from evaluation by the institutional review board of Korea University because it is an ecological study using publicly available data.

### 2.2. Dependent Variables: Suicide Rates 

The dependent variable was the age-adjusted suicide rate in the previous five years (2010–2014). The Ministry of Statistics provides crude and age-adjusted mortality based on National Death Registration, which is census data providing cause of death by the International Classification of Diseases 10th Version (ICD-10 version), and basic sociodemographic information and National census data, providing demographic information on the South Korean population. Suicide mortality was defined when the cause of mortality was coded within the range from X60 to X84 by the ICD-10 version. Age-adjusted mortality using the 2005 census population as the standard was used in this study.

### 2.3. Independent Variables: Risk Factors 

We used six independent variables: single household, divorce rate, financial independence, social welfare budget, depression and subjective health status. As sociodemographic variables, single household and divorce were used. Regarding economic and public services, financial independence and the social welfare budget were used. Health related factors were analyzed using depression and subjective health status variables taken from a community health survey. First, we defined sociodemographic factors as the ratio of single households to the total number of households and divorce rates to the number of divorces among the total population. Second, for economic and public service factors, financial independence was defined by the ratio of nontax revenue and local taxes for regional general accounting, which shows the economic status of each area. The social welfare budget is defined as the ratio of the social welfare budget used in regional general accounting. Third, for health-related factors, depression was measured by responses (yes or no) to the following question: “In the past year, have you felt depressed for two consecutive weeks?”, while Subjective health status was measured by the ratio of responses (very good and good) to subjective health status questions. We assumed that there would be lag effect on suicide rates, i.e., after experiencing risk factors for 1 year to 5 year, people may commit suicide. In this study, we used risk factor variables from 2010. A summary of all variables is presented in Table 1.

### 2.4. Statistical Analysis

We used descriptive statistics to identify general characteristics of maximum, minimum, average, and standard deviation. To identify regional variation in suicide rates, we used Extremal quotient (EQ) and Coefficient of variance (CV). EQ was calculated by the ratio of the maximum to the minimum value. CV was average divided by standard deviation. EQ is widely used to determine variations in medical procedures such as surgical procedures and examination. However, some research has used EQ and CV to determine variations in the prevalence and incidence of diseases such as hypertension and diabetes.

For bivariate analysis, Pearson’s correlation analysis was performed using suicide rates and risk factors. Correlation coefficients and variance inflation factor (VIF) are used by statisticians and epidemiologists [32]. Even though a VIF higher than 10 and a correlation coefficient cut off greater than 0.8 are common in measuring multicollinearity [33], a VIF of between 5 or 10 and a correlation coefficient cutoff higher than 0.5 are suggested [34,35].

Finally, the multiple linear regression model was applied to estimate associations between suicide rates and potential risk factors using single household, divorce rate, financial independence, social welfare budget, depression, and subjective health status [14,36]. The SPSS software (version 24.0) (SPSS Inc., Chicago, IL, USA) was used for data analyses. Statistical significance was verified by a two-tailed test and *p*-values of 0.05 were the threshold of this analyses.

## 3. Results

The general characteristics (suicide rate, single household rate, financial independence rate, social welfare budget, divorce rate, depression prevalence, subjective health status) of the 252 regions are presented in Table 2. The average suicide rate was 142.7 per 100,000 population. The average single household was 22%, and financial independence was 30%. The average social welfare budget was 26%. The average divorce rate was 2.2%. The average depression prevalence was 5.11%. The average subjective health status was 49%. The EQ of suicide rate was 2.88, which indicates regional variation in Korea. Among the independent variables, depression showed the greatest variation among risk factors (EQ = 55.00), followed by social welfare budget (13.05), financial independence (9.94), single household (3.25), divorce rate (3.18), subjective health status (2.04).

In a Pearson’s correlation analysis in Table 3, financial independence, social welfare budget and divorce rate were associated with suicide rates. Regarding economic and public service factors, financial independence (*r* = −0.435, *p* < 0.01), and social welfare budget (*r* = −0.385, *p* < 0.01) showed stronger associations than sociodemographic factors, e.g., divorce rate (*r* = 0.178, *p* < 0.01). Health related variables were not significantly associated with suicide rate. Correlations of all variables were less than 0.7, which proves that there is independence within variables. Also, using tolerance and VIF, we verified multicollinearity. Since tolerance was higher than 0.2 and VIF was less than 10, there was no multicollinearity among variables.

Table 4 shows the associations between suicide rate and risk factors at the regional level. Financial independence was associated with suicide rate at an area level (*b* = −0.662, *p* < 0.001), as was Social welfare budget (*b* = −0.754, *p* < 0.001). Divorce rate was associated with suicide rate at the regional level (*b* = 17.743, *p* < 0.001). Economic and public service factors and sociodemographic factors were associated with suicide rate after controlling other variables. Health-related variables were not significantly associated with suicide rate. To compare the degree of association, we used standardized b, which adjusts risk factors for the same condition. The strongest risk factor for suicide was shown to be financial independence (0.463), followed by social welfare budget (0.370) and divorce rate (0.282).

## 4. Discussion

We found that there is a regional variation in suicide rates in South Korea. Financial independence, social welfare budget and divorce rates were associated with suicide rates at the regional level, even after controlling other variables. To the best of our knowledge, no study has presented EQ regarding suicide rates at the regional level. Here, we show the EQ indexes from some previous studies. In Taiwan, for example, EQ was 15.68, and in England, it was 74.07, i.e., far higher than in of South Korea. These EQ values are not applicable to South Korean because each country differs in size and has different definitions of “region”. On the other hand, we could compare the EQ of suicide with other causes of death in South Korea. Statistics Korea provides the top 10 cause of death every year. In 2014, malignant neoplasm was responsible for the highest mortality, followed by heart diseases, cerebrovascular diseases, pneumonia, suicide, diabetes, chronic lower respiratory diseases, liver diseases, traffic accidents and hypertension. Compared with these causes of death, suicide has the third lowest EQ value, following malignant neoplasm and cerebrovascular diseases.

We found that financial independence was correlated with suicide mortality rates. Financial independence represents the economic status of administrative governments. Low financial independence implies financial deficit or austerity. Karanikolos et al. reviewed financial crises and health in Europe, and showed that financial difficulties among governments led to epidemics of suicide [37]. Another study in Europe showed that economic crises (worsening employment and gross domestic product) correlated with suicide rates [38]. In Greece, government austerity policy (public expenditure reduction) due to budget cuts led to increases in suicide [39].

We found that the social welfare budget is a strong predictor of suicide rates. In the present study, the social welfare budget was negatively correlated with suicide rates; some studies showed the same results in the U.S [40,41,42]. These studies compared suicide rates and public spending/expenditure in each state [40,41]. Social welfare supports people who are facing financial problems such as unemployment or underemployment [40]. The magnitude of the social welfare budget indicates that the government makes efforts to provide social welfare services to citizens [40].

Divorce rates were associated with suicide rates; with increase in divorce rates, suicide rates increased. This correlation has been observed in other studies [43,44]. Even though divorce is legal, it has a great impact on the economy and the community; e.g., people who are divorced experience changes in income and in social interactions [45]. In addition, divorced people report higher social isolation than married people [46]. These results were supported by the theory of Durkheim. According to the Durkheim, divorce can break family ties, which contributes to lower social integration, and egoistic suicide [16]. Moreover, divorce also increase anomic suicide [44].

We did not find any association between health-related factors and suicide rate at the regional level. In general, depression and subjective health status appear to be factors related to suicide rates at the individual level, but this tends to be unclear in ecological studies conducted at the regional level. An ecological study in the United States indicated that major depression was not related to suicide rates [47], and a Korean ecological study found that subjective health levels were not related to suicide rates [48]. This was probably because depression and subjective health status are accurately measured at the individual level, so they are very limited in terms of accurately reflecting regional characteristics, partly due to small sample sizes. Nonetheless, some studies which observed a relationship between regional characteristics and suicide rates excluded factors measured at the individual level [49,50,51].

This study has several limitations. First, since it is an ecological study, the ecological fallacy should be kept in mind. Second, we did not include age groups and gender as risk factors. In South Korea, suicide rates are far higher among males than females, and among old adults (i.e., more than 65 years). Third, more varied and accurate variables are needed. We used single household and divorce rate as demographic factors, financial independence and social welfare budget as economic and public service factors, and depression and subjective health status as health related factors. Future studies need to include indicators such as Gini’s coefficient, deprivation index and relative index of inequalities. Also, alcohol or substance abuse needs to be considered. Substance abuse disorder has been reported as an important risk factor for suicide in South Korea [52,53]. Forth, various methodologies should be used to achieve more refined results. Some methodologies have been developed which could be applied to such a study, such as geographic weighted regression and the Bayesian hierarchy model [20,27].

## 5. Conclusions

The present study revealed that there is substantial variation in suicide rates among South Korean regions. In addition, there are relationships between suicide mortality rates and various factors such as sociodemographic, economic, public service and health-related factors. Financial independence and social welfare budget were strong risk factors for suicide at the regional level. Adequate social policy, strengthening financial independence and social welfare budget should be more closely examined. These societal circumstances should be noted in further studies of suicide etiology and prevention.

## Figures and Tables

**Table 1:** Variables and their definitions.

Type	Name	Definition
Dependent Variable	Suicide Rate	X60–X84 (ICD-10)
Independent variable	Single household	Ratio of Single households
	Divorce rate	Ratio of divorce
	Financial independence	Ratio of local tax and nontax revenue for regional general accounting
	Social welfare budget	Ratio of social welfare budget for regional general accounting
	Depression	Ratio of depression lasting 2 weeks in the past year
	Subjective health status	The ratio of “very good” and “good” in subjective health status

**Table 2:** Variations in suicide mortality and independent variables.

Variables	Minimum	Maximum	Average	EQ	CV
Suicide mortality rate	75.5	217.8	142.7	2.88	18.70
Single household	12.2	39.6	22.8	3.25	20.96
Financial independence	8.6	82.9	30.4	9.94	58.35
Social welfare budget	4.56	59.52	26.4	13.05	47.03
Divorce rate	1.1	3.5	2.2	3.18	17.71
Depression	0.2	11	5.1	55.00	42.80
Subjective health status	35.5	72.3	49.2	2.04	14.00

EQ: Extremal quotient; CV: Coefficient of variance.

**Table 3:** Correlation between suicide rate and independent variables.

Variables	Single Households	Divorce Rate	Financial Independence	Social Welfare Unit	Depression	Subjective Health Status
Suicide mortality rate	−0.001	0.178 **	−0.435 **	−0.385 **	−0.101	0.050
Single household	1	0.033	−0.123	0.133 *	−0.047	0.040
Financial independence	0.033	1	0.187 **	0.100	0.092	−0.169 **
Social welfare budget	−0.123	0.187 **	1	0.143 *	0.217 **	−0.100
Divorce rate	0.133 *	0.100	0.143 *	1	0.213 **	−0.185 **
Depression	−0.047	0.092	0.217 **	0.213 **	1	−0.520 **
Subjective health status	0.040	−0.169 **	−0.100	−0.185 **	−0.520 **	1

*: *p* < 0.05, **: *p* < 0.01.

**Table 4:** Associated factors in regional variations in suicide rates.

Variables	*b*	SE	Standardized *b*	95% Confidence	*p*-Value
(constant)	135.765	16.510		103.244–168.285	<0.001
Single household	−0.081	0.270	−0.015	−0.613–0.452	0.766
Financial independence	−0.662	0.075	−0.463	−0.810–−0.514	<0.001
Social welfare budget	−0.754	0.107	−0.370	−0.963–−0.544	<0.001
Divorce rate	17.743	3.243	0.282	11.356–24.130	<0.001
Depression	0.970	0.706	0.083	−0.420–2.361	0.170
Subjective health status	0.082	0.218	0.022	−0.346–0.511	0.706
